# How do private practitioners in Pakistan manage children suspected having tuberculosis? A cross sectional study

**DOI:** 10.1186/s12889-020-10053-4

**Published:** 2021-01-07

**Authors:** Aashifa Yaqoob, Sven Gudmund Hinderaker, Razia Fatima, Hina Najmi, Anwar-ul-Haq

**Affiliations:** 1 Common Management Unit (HIV/AIDS, TB & Malaria), Block F, EPI Building, Near National Institute of Health (NIH) (Prime Minister’s National Health Complex), Park Road, Islamabad, Pakistan; 2 University of Bergen, Bergen, Norway; 3 Health Services Academy, Islamabad, Pakistan; 4 Ministry of National Health Services Regulation & Coordination, Government of Pakistan, Islamabad, Pakistan

**Keywords:** Children, Tuberculosis, Private providers, Management practices, Referral, Diagnosis, Presumptive, Pakistan

## Abstract

**Background:**

In Pakistan, private providers provide a large portion of health care, including for tuberculosis (TB). All TB patients are supposed to be reported to the National Tuberculosis Program (NTP), which provides drugs free of charge in addition to monitoring, supervision, and support. However, diagnosis of TB in children is difficult. We aimed to assess the private health care providers’ investigation practices and management of childhood TB.

**Methods:**

We used a cross-sectional study, which was based on a national survey measuring under-reporting of children with TB in 12 selected districts in Pakistan from April–June, 2016. We explored the practices of the private health care providers, including the health care workers i.e. general practitioners, pediatricians, pulmonologists and chest specialists, who were involved in the diagnosis of TB in children under 15 years for investigating and managing children suspected having TB.

**Results:**

Among 6519 presumptive child TB cases, a total of 5193(79.7%) children under 15 years were diagnosed as TB by private health care providers during second quarter, 2016. Only 187(2.9%) were notified to NTP. The majority of presumptive child TB cases reported cough, fever, and failure to thrive; few had TB contacts with pulmonary TB patients. Failure to thrive, loss of body weight and absence of BCG (Bacillus Calmette–Guérin) scar was more common in female children. Private providers relied on chest X-ray in 46.1%, while tuberculin skin test and Gene-Xpert MTB/RIF testing was little utilized. Bacteriological confirmation was present in 7.6%, and clinical assessment was the only basis for diagnosis in 39.3%. Of children with presumptive TB, only 955(14.6%) children were treated by private provider, while 3121(47.9%) cases were referred for diagnosis and 2443(37.5%) were referred after diagnosis for treatment; among all the referred, 3812(68.5%) were sent for investigations to District TB Centre (NTP).

**Conclusion:**

This study showed that many private providers referred children suspected having TB to laboratories for further diagnosis, but the cases identified in these investigations were often not notified to the NTP. This problem could be resolved by strengthening the referral linkages between private health providers, NTP laboratories and treatment centres through capacity building and training of their staff.

## Background

Tuberculosis (TB) among children is a significant global challenge affecting mainly low- and middle income countries. In 2018, 1.1 million children fell ill with TB, and 205,000 (18%) of them died [1]. Of the childhood cases, 75% occur in 22 high-burden countries that together account for 80% of the world’s estimated incident cases [2, 3]. In terms of global TB control measures, children have a lower priority because they are considered to be less contagious and, therefore, a less important source of infection [3]. Globally, childhood TB cases are under-reported. This is probably due to the difficulty of confirming the diagnosis. Some of the challenges related to assessing the actual magnitude of TB in children include poor implementation of the national guidelines, inappropriate diagnoses, inadequate drug regimens and lack of knowledge about case management [4]. As a notifiable disease in most countries, all diagnosed cases must be recorded and reported.

**Fig. 1:**
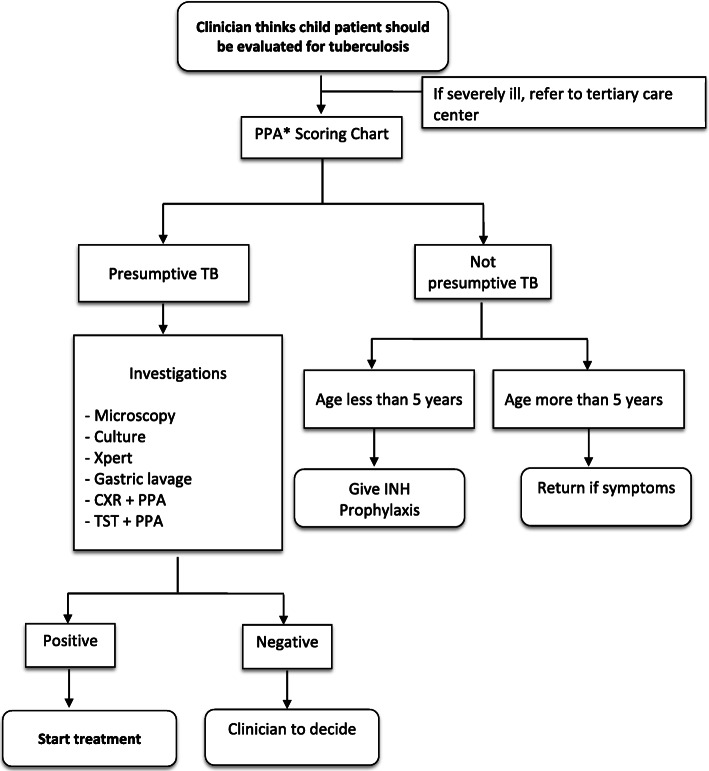
NTP Flow chart for evaluation of a child with suspected TB. *PPA = Pakistan Paediatric Association

In Pakistan, it is estimated that over 562,000 people were infected with TB in 2018, of whom 369,548 TB cases were notified; and among these 13% were children under 15 years. Private health care providers in Pakistan contributed 32% of all TB notifications [1]. During 2006–07, NTP Pakistan developed its national childhood TB policy guidelines in collaboration with Pakistan Pediatric Association (PPA), aiming to facilitate the pediatricians, physicians and other health workers to improve and standardize clinical decisions for investigating presumptive child TB cases (< 15 yrs) in Pakistan. In these guidelines, a score chart evaluates the likelihood of pulmonary and extra pulmonary TB based on clinical, histological and radiological features [5]. Currently, the PPA score chart is recommended by NTP to all pediatricians to help diagnose children with suspected TB, when presenting with prolonged or unexplained illness of more than 2 weeks. A flow chart for evaluation of a child with suspected TB is given in Fig. 1.

A recent patient-pathway analysis in Pakistan confirmed the important role of the private sector in providing TB care in Pakistan, and highlighted the extent of utilization of private sector (85%) by the patients as entry points to care [6]. This is crucial for understanding the role of the private sector in the diagnosis and treatment of pediatric TB, but limited evidence is available on this issue [7, 8]. Therefore, we aimed to assess the practices of private health care providers in investigation of children suspected having TB. Our target group was patients under 15 suspected having tuberculosis in Pakistan, and our specific objectives were 1) to assess signs and symptoms that private health providers record; and 2) to assess investigation and referral practices.

## Methodology

### Design

This is a descriptive cross sectional study based on a national child TB inventory involving a surveillance system that was established among all non-NTP facilities in a sample of 12 districts across Pakistan from April–June, 2016 [9].

### Setting

Pakistan is the sixth most populous country of the world with an estimated population of 208 million in 2017. Approximately 64% live in rural areas [10]. The public sector is the main source of preventive health care, and has primary, secondary and tertiary levels of care. Quality-assured diagnosis and treatment for TB is provided by NTP free of charge to patients through general facilities in all public and selected private sector facilities. TB services in Pakistan are integrated into the primary health care system at the district level, and are coordinated at this level by the district TB coordinator who is responsible for monitoring, supervising and supporting all clinics in the given district. The data from the districts is monitored and evaluated at provincial and national levels. Patients are reported from rural health centres and sub district hospitals to district hospitals, where the TB coordinator is usually based. Other large private clinics engaged with NTP should report cases directly to Provincial TB Program.

Wealth distribution in Pakistan is highly skewed, with a larger proportion of lowest wealth quintile living in rural settings. In rural Punjab, 18% of population are in the lowest quintile, while these account for only 1% in urban Punjab. In rural Sindh, 69% are in the lowest quintile, while only 6% live in urban Sindh. In rural KPK (Khyber Pakhtunkhwa), 20% are in lowest quintile, while 2.3% live in urban KPK. In rural AJK (Azad Jammu & Kashmir), 15% are in lowest quintile, while 2% live in urban AJK. In rural Balochistan, 36% are from the lowest quintile, while in urban Balochistan 11% are from the lowest quintile [11].

### Study site

We selected 12 districts in Pakistan, with representation from all four provinces (Balochistan, KPK, Punjab and Sindh) and two regions (GB (Gilgit Baltistan) and AJK). The selection of the districts was partly based on needing to have a sample proportional to the population size of the children [9]. Districts where security is an issue were excluded from the sampling process. The study was carried out in all private health facilities that manage childhood TB in the 12 selected districts across Pakistan [9]. All non-NTP private facilities in the selected districts were mapped and consenting private health providers managing childhood TB in 12 districts across Pakistan were enrolled. Non-NTP private facilities refer to private facilities that have no formal collaboration with NTP.

### Study population

The study population was all children brought to a clinician at a non-NTP private facility who considered tuberculosis a potential diagnosis, because of a prolonged or unexplained illness lasting more than 2 weeks. All participants were identified by health care providers who were not engaged with the NTP i.e. general practitioners, pediatricians, pulmonologists and chest specialists who were involved in child TB inventory study April – June, 2016. Of all the health care providers who were mapped and invited to participate in the study, 82% agreed.

### Data collection

A register for presumptive child TB cases was introduced to health facilities diagnosing childhood TB in order to record all information regarding history of presumptive TB cases as well as to facilitate investigation and management. All of the health care providers who consented to participate in the study were briefed on how to capture the required information in the registers. Immediately following these instructions, and without direct mentoring but with close follow up, the health care providers undertook the data collection for the period of 3 months (second quarter of 2016) and the management of child TB patients by the non-NTP health care providers was recorded without having them change their routine practice. To improve the accuracy and validity of the data, a mobile based data collection tool was used in this survey [12]. Data entry was done directly on site on mobile phones using an application developed by “Zong 4G (Mobile network operator company)”. Field officers were provided mobile phones to enter the data when visiting the health facilities on a weekly basis. In addition, participants were visited every 2 weeks by the district TB coordinators along with a provincial coordinator, a supervisor and field officer to ensure the quality of data collected (completeness, correctness) for accurate record linkage, and for cross-checking the status of the NTP registration.

### Variables and data collection tools

The data collection tool was based on all information regarding the diagnosis and management practices by private healthcare providers concerning children with presumptive TB. Variables included age, sex, place, symptoms, investigations, recommendations given, and referral decision. Data quality auditing of every record was conducted to ensure the validity of data by crosschecking from the hard copies.

### Data analysis

Descriptive statistics were used to summarize the investigation, management and referral of children with presumptive TB by private providers. Cross tabulation was done to identify any differences between children 0–4, 5–11, and 12–14 years. Analyses were done in STATA version 14.

## Results

Table 1 shows that 5193 children were diagnosed as having TB in 12 selected districts of various population sizes. Many doctors (37.5%) referred diagnosed TB cases to NTP for further management, but few notified NTP if they initiated treatment themselves. There was great variation between the districts in terms of referral rates (2.3–76.1%) and notifications (0–18.5%) of the child TB cases.

**Table 1:** Presumptive tuberculosis patients under 15 years identified by private health providers in selected districts in Pakistan, 2016

Province	District	Population (< 15 yrs) N	CNR^a^ 2016 per 100,000	Presumptive child TB cases n	Diagnosed TB^a^ n (%)	Referred to NTP^a^ n (%)	Notified to NTP (Project notification) ^a^ n (%)
All sites		8,643,221	193	6519 (0.08)	5193 (79.7)	2443 (37.5)	187 (2.9)
Punjab	Attock	483,575	175	497 (0.10)	442 (88.9)	97 (19.5)	34 (6.8)
	Chiniot	362,756	232	671 (0.18)	337 (50.2)	139 (20.7)	4 (0.6)
	Hafizabad	317,804	219	874 (0.28)	555 (63.5)	32 (3.7)	12 (1.4)
	Vehari	837,748	225	376 (0.05)	333 (88.6)	223 (59.3)	7 (1.8)
Sindh	Shikarpur	390,208	126	623 (0.16)	394 (63.2)	128 (20.5)	2 (0.3)
	Hyderabad	650,492	149	838 (0.13)	838 (100)	638 (76.1)	35 (4.2)
	Karachi	4,366,147	143	1041 (0.02)	942 (90.5)	737 (70.7)	12 (1.2)
KPK^a^	Buner	211,496	157	232 (0.11)	148 (63.8)	89 (38.4)	43 (18.5)
	Peshawar	843,278	244	1034 (0.13)	999 (96.6)	340 (34.0)	36 (3.5)
AJK^a^	Pallundary	97,553	89.2	118 (0.12)	114 (96.6)	15 (12.7)	2 (1.7)
Balochistan	Jhal Magsi	46,011	29	44 (0.10)	27 (61.4)	1 (2.3)	0 (0.0)
GB^a^	Ghizer	36,153	133	171 (0.47)	64 (37.4)	4 (2.3)	0 (0.0)

^a^ **Footnotes:** *KPK* Khyber Pakhtunkhwa, *AJK* Azad Jammu & Kashmir, *GB* Gilgit Baltistan, *CNR* Case Notification Rate (Routine Notification to NTP all forms)

“Diagnosed TB by PP” with proportion out of all presumptive patients (“yield”). “Referred to NTP” with proportion out of all diagnosed patients. “Notified to NTP” with proportion out of all presumptive child TB patients

Table 2 shows the signs and symptoms of the children suspected having TB, by age group. We noticed many had coughs (92.1%), fever (89.0%), and failure to thrive (64.8%). Few reported contacts with a TB patient (11.9%). A BCG-scar was absent in 19.6% of children 0–4, 28.3% of children 5–11 and 37.4% of children 5–14, P = <.0001.

**Table 2:** Signs and symptoms of children with presumptive tuberculosis by age groups, recorded by private health care providers in 12 selected districts in Pakistan, 2016

History and investigations		Total	0–4 years	5-11 years	12-14 years	***p***-value
		n(%)	n(%)	n(%)	n(%)	
Total	All presumptive cases	**6519 (100)**	**1691 (100)**	**3214 (100)**	**1614 (100)**	< 0.0001
	Girls	2320 (35.6)	639 (37.8)	1063 (33.1)	618 (38.3)	
	Boys	4199 (64.4)	1052 (62.2)	2151 (66.9)	996 (61.7)	
Chest	Cough more than two weeks	6006 (92.1)	1560 (92.3)	2959 (92.1)	1487 (92.1)	0.974
	Failure to thrive	4210 (64.8)	1129 (66.8)	1947 (60.6)	1134 (70.3)	< 0.0001
Systemic	Fever	5794 (89.0)	1538 (91.0)	2836 (88.2)	1420 (88.0)	0.038
	Loss of body weight	504 (7.7)	115 (6.8)	237 (7.4)	152 (9.4)	0.011
	Enlarged cervical lymph nodes	785 (12.1)	147 (8.7)	389 (12.1)	249 (15.4)	< 0.0001
	BCG scar absent	1843 (28.2)	331 (19.6)	908 (28.3)	604 (37.4)	< 0.0001
Meningitis	Signs of slow onset meningitis^a^	658 (10.1)	115 (6.8)	330 (10.3)	213 (13.2)	< 0.0001
Contacts	Known pulmonary tuberculosis patient	778 (11.9)	234 (13.8)	391 (12.2)	153 (9.5)	< 0.0001

^a^Symptoms regarded as slow meningitis include headache, vomiting, irritability, lethargy, neck stiffness, bulging fontanella, coma

Table 3 shows signs and symptoms of children suspected having TB, by gender. Out of 6519 presumptive child TB cases, 6006 (92.1%) had reported having a history of coughing for more than two weeks i.e. 3904 (93.0%) males and 2102 (92.1%) females. Of the girls, a BCG-scar was absent in 742 (32.0%) compared to 1101 (26.2%) boys (*P* < 0.0001). Moreover, the differences in coughing, failure to thrive, enlarged lymph nodes and absence of BCG scar in male and female children were statistically significant (*P* > 0·05).

**Table 3:** Signs and symptoms of children with presumptive tuberculosis by gender, recorded by private health care providers in 12 selected districts in Pakistan, 2016

History and investigations		Total	Female	Male	p-value
		n(%)	n(%)	n(%)	
Total	All presumptive cases	**6519**	**2320**	**4199**	
Chest	Cough more than two weeks	6006 (92.1)	2102 (90.6)	3904 (93.0)	0.001
	Failure to thrive	4210 (64.8)	1644 (70.9)	2566 (61.1)	< 0.0001
Systemic	Fever	5794 (89.0)	2039 (87.9)	3755 (89.4)	0.075
	Loss of body weight	504 (7.7)	196 (8.4)	308 (7.3)	0.110
	Enlarged cervical lymph nodes	785 (12.1)	322 (13.8)	463 (11.0)	0.001
	BCG scar absent	1843 (28.2)	742 (32.0)	1101 (26.2)	< 0.0001
Meningitis	Signs of slow onset meningitis^a^	658 (10.1)	228 (9.8)	430 (10.2)	0.607
Contacts	Known pulmonary tuberculosis patient	778 (11.9)	258 (11.1)	520 (12.4)	0.121

^a^Symptoms regarded as slow meningitis include headache, vomiting, irritability, lethargy, neck stiffness, bulging fontanella, coma

Table 4 shows investigations done on the 6519 children suspected having TB, by age group; 1564 (92.4%) children under five, 2545 (79.2%) children 5–11 and 1084 (67.2%) were diagnosed with tuberculosis: 4695 (72.0%) clinically and 498 (7.6%) bacteriologically verified. The most common investigation was chest X-ray (46.1%). Sputum smear was done on 14.3% among participants 0–4 years, 28.3% among participants 5–11 years, and 723 (44.8%) among 12–14 years. Clinical assessment was the only investigation done on 49.8% of the children below 5 and bacteriological confirmation was more in children 5–11 years (6.3%) and children 12–14 (14.0%), < 0.0001. Many children with presumptive TB were referred to the district TB centre for diagnosis and treatment: 62.3% of children 0–4 years, 69.9% of children 5–11 and, 71.8% of children 5–14. Few children were notified to the NTP (187 children, 2.9%), more girls than boys (4.9% vs.1.8%,). Out of the girls referred for diagnosis, patients notified to NTP were 16 (14.2%) 0-4y, 54 (47.8%) 5-11y, and 43 (38.1%) 12-14y. Among the boys referred for diagnosis those notified were 19 (25.7%) 0-4y, 36 (48.6%) 5-11y, and 19 (25.7%) 12-14y.

**Table 4:** Management by private practitioners of children with presumptive tuberculosis, by age groups, in 12 selected districts in Pakistan, 2016

Practice of private health care providers		Total	0–4 years	5-11 years	12-14 years	p-value
		n(%)	n(%)	n(%)	n(%)	
Presumptive child TB		**6519**	**1691**	**3214**	**1614**	
Diagnosed TB	All	5193 (79.7)	1564 (92.4)	2545 (79.2)	1084 (67.2)	< 0.0001
	Bacteriologically positive	498 (7.6)	68 (4.0)	204 (6.3)	226 (14.0)	
	Clinically diagnosed	4695 (72.0)	1496 (88.5)	2341 (72.8)	858 (53.1)	
Notified	Notified to NTP	187 (2.9)	35 (2.1)	90 (2.8)	62 (3.8)	< 0.0001
Investigation practices	Tuberculin Skin/PPD testing	219 (3.4)	45 (2.7)	112 (3.5)	62 (3.8)	0.1464
	Sputum Smear	1875 (28.8)	242 (14.3)	910 (28.3)	723 (44.8)	< 0.0001
	X-ray	3005 (46.1)	683 (40.4)	1499 (46.6)	823 (51.0)	< 0.0001
	X-pert test	324 (5.0)	34 (2.0)	146 (4.5)	144 (8.9)	< 0.0001
	Granuloma/Histopathology	837 (12.8)	175 (10.4)	406 (12.6)	256 (15.9)	< 0.0001
	Culture	152 (2.3)	13 (0.8)	73 (2.3)	66 (4.1)	< 0.0001
Number of tests done^*^	Only clinical assessment	2559 (39.3)	842 (49.8)	1219 (37.9)	498 (30.9)	< 0.0001
	1 test	2217 (34.01)	574 (33.9)	1165 (36.3)	478 (29.6)	
	2 tests	1270 (19.5)	223 (13.2)	617 (19.2)	430 (26.6)	
	3 tests or more	473 (7.3)	52 (3.1)	213 (6.6)	208 (12.9)	
Management practices	Referred for TB diagnosis	3121 (47.9)	842 (49.8)	1578 (49.1)	701 (43.4)	< 0.0001
	Diagnosed and referred	2443 (37.5)	494 (29.2)	1293 (40.2)	656 (40.6)	
	Treated	955 (14.6)	355 (21.0)	343 (10.7)	257 (15.9)	
Place of referral (*n* = 5564)	District TB Centre (NTP)	3812 (68.5)	832 (62.3)	2006 (69.9)	974 (71.8)	< 0.0001
	Private laboratory	1190 (21.4)	388 (29.0)	561 (19.5)	241 (17.8)	
	Private specialist hospital/GP	562 (10.1)	116 (8.7)	304 (10.6)	142 (10.5)	

*indicates how many tests were performed to reach final diagnosis

The management practices of child TB patients stratified by gender is given in Table 5. Out of presumptive cases, 80.6% of girls and 79.2% of boys were diagnosed with TB. Bacteriological confirmation was noted for 244 girls and 254 boys, but the proportion of bacteriologically positives out of all the suspected cases was higher among girls (10.5%) than boys (6.0%). The NTP was notified about only a few of the children (187 children, 2.9%), with more girls than boys (4.9% vs.1.8%,). A higher proportion of girls (35.8%) with presumptive TB than boys (24.9%) were examined for sputum smear. However, referral for diagnosis was more common for boys (50.6%) than girls (43.0%).

**Table 5:** Management by private practitioners of children with presumptive tuberculosis, by gender, in 12 selected districts in Pakistan, 2016

Practice of private health care providers		Total	Female	Male	p-value
		n(%)	n(%)	n(%)	
Presumptive child TB		**6519**	**2320**	**4199**	
Diagnosed TB	All	5193 (79.7)	1869 (80.6)	3324 (79.2)	< 0.0001
	Bacteriologically positive	498 (7.6)	244 (10.5)	254 (6.0)	
	Clinically diagnosed	4695 (72.0)	1625 (70.0)	3070 (73.1)	
Notified	Notified to NTP	187 (2.9)	113 (4.9)	74 (1.8)	< 0.0001
Investigation practices	Tuberculin Skin/PPD testing	219 (4.7)	106 (4.6)	113 (2.7)	< 0.0001
	Sputum Smear	1875 (28.8)	830 (35.8)	1045 (24.9)	< 0.0001
	X-ray	3005 (46.1)	1127 (48.6)	1878 (44.7)	0.003
	X-pert test	324 (5.0)	154 (6.6)	170 (4.1)	< 0.0001
	Granuloma/Histopathology	837 (12.8)	331 (14.3)	506 (12.1)	0.010
	Culture	152 (2.3)	67 (2.9)	85 (2.0)	0.03
Number of tests done*	Only clinical assessment	2559 (39.3)	844 (36.4)	1715 (40.8)	< 0.0001
	1 test	2217 (34.01)	699 (30.1)	1518 (36.1)	
	2 test	1270 (19.5)	543 (23.4)	727 (17.3)	
	3 tests or more	473 (7.3)	234 (10.1)	239 (5.7)	
Management practices	Referred for TB diagnosis	3121 (47.9)	997 (43.0)	2124 (50.6)	< 0.0001
	Diagnosed and referred	2443 (37.5)	857 (36.9)	1586 (37.8)	
	Treated	955 (14.6)	466 (20.1)	489 (11.7)	
Place of referral (n = 5564)	District TB Centre (NTP)	3812 (68.5)	1260 (68.0)	2552 (68.8)	0.136
	Private laboratory	1190 (21.4)	386 (20.8)	804 (21.7)	
	Private specialist hospital/GP	562 (10.1)	208 (11.2)	354 (9.5)	

*indicates how many tests were performed to reach final diagnosis

## Discussion

Our study found that almost half of the private health care providers investigating children for TB had used chest X-ray. Once suspected for TB, many were diagnosed (79.7%). Many doctors referred presumptive TB cases to NTP for further diagnosis and management. Private doctors who started TB treatment rarely (2.9%) reported the cases to NTP if they initiated treatment themselves.

This study indicated that the diagnosis of childhood TB by private providers was mainly based on clinical features, radiography and microscopy, and rarely on tuberculin skin tests, histopathology and Gene- Xpert MTB/RIF. Results from other settings has also shown that TB diagnosis in children is often based on a combination of clinical symptoms and chest X-ray; this could be due to the lack of a simple and precise diagnostic tool, especially at the local level, or due to inadequate training and capacity of health care workers [8, 13–16]. In Pakistan, the availability of diagnostic tools varies across the country. Chest X-ray sand smear microscopy are almost universally available and used for TB diagnosis at peripheral levels. Histopathology, tuberculin skin test, sputum culture and Gene-Xpert MTB/RIF are only available at tertiary care hospital laboratories. Gene-Xpert MTB/RIF testing of patient stools has been shown to be a useful technique for identifying children with TB [17], and could be a good addition to traditional tests. However, in Pakistan the limited availability of such tests in rural areas makes it currently less universal.

An important finding of the study was that private health care providers referred many children with presumptive TB: 3121 (47.9%) for diagnosis and 2443 (37.5%) for treatment. They only initiated treatment in 14.6% of the diagnosed cases. Of all the referred presumptive TB cases, 3812 (68.5%) were referred for diagnosis to district NTP centres. However, only 2.9% of the referred cases were registered in the NTP registers. This large gap in reporting treatment outside the NTP system could be due to several factors: poor interdepartmental coordination between the laboratory and the treatment centres; inadequate counselling of presumptive TB patients by the laboratory technicians; and weak referral mechanisms [18–21]. The communication between laboratories and treatment centres could be improved by having regular weekly visits by district health coordinator to the laboratories, and by contacting the referring private doctor to discuss further management of cases according to the NTP guidelines. Across Pakistan, treatment services are also available in the public facilities that have diagnostic capacity. It has been reported that there may be a lack of trust in public sector to provide quality care, and thus few patients sought care in the public sector [6]. It is also possible that some referred TB patients might not actually go to NTP, and perhaps received treatment in the private sector. A similar finding is also reported in a study from Indonesia, where only 2% of childhood TB cases recorded in hospitals were reported to the NTP [22]. In Pakistan, childhood TB is managed by various providers and various levels of the health care sector. There is an urgent need to improve communication between the NTP and other health care providers by increasing engagement in the private sector through training and capacity building on the national guidelines for managing childhood TB cases [23]. For example, mHealth could potentially accelerate TB notification from the part of private sector that is not collaborating with NTP [24, 25].

Almost all children had coughs and fever, and most had failure to thrive, which is consistent with the guidelines [23]. BCG vaccination is associated with decreased severity of tuberculosis [26] and BCG is part of the child immunization program in Pakistan A lack of a BCG scar was more common in older children, which may reflect the improved Expanded Program on Immunization (EPI) performance from 2012 to 2018. The percentage of fully immunized children aged 12–23 months increased from 54% in 2012–13 to 66% in 2017–18 [11]. Vaccination coverage inequalities exist at sub district levels, ranging from 58 to 85% in rural to urban areas and from 60 to 80% in lower to higher income quintiles [27].

In this study, we found that a higher proportion of adolescents reported respiratory symptoms, underwent sputum testing, and had bacteriological confirmation. Adolescents are important for TB control and can contribute to substantial transmission in settings such as schools. WHO suggests efforts to develop integrated family- and community-centered strategies to provide comprehensive and effective services at the community level to improve child and adolescent notification [28]. Another potential reason for this higher proportion is that adolescents are easier to test for sputum than younger children.

This study showed that failure to thrive and loss of body weight was more common in girls. This can be partly a biological difference and effect of culture and nutrition [29]. A study in India showed that the dietary intake of energy, iron, calcium and protein was significantly higher in boys than girls [30]. The slightly higher absence of BCG scar in girls could be explained by less care for girls in Pakistan, where a boy is usually more valued than a girl [31]. Similar differences in non-utilization of child immunization are reported elsewhere [32, 33].

Our study had several strengths. A major strength of this study is the large total sample with participants from all provinces, and we believe it may reflect the diverse situation in this country. In this study, validity of the data was ensured though data quality audit by crosschecking every record from the hard copies to remove inconsistencies. Also using mobile phone for data collection reduced data entry errors by eliminating one step for database creation. This study adheres to “STROBE” guidelines for observational studies [34, 35].

The study also had some limitations. Although it had a large total sample, the number of clusters was limited to the number of provinces, giving lower precision. Despite this, it probably reflects fairly well the different situations in the country. Also, our study did not include actual observations through field assessments. so the accuracy and completeness of the data could therefore not be totally ensured. High referral to NTP centres for diagnosis may be partly because the study was closely related to NTP, and data collectors from NTP visited the study sites twice a month, and this could affect reporting, like a Hawthorne effect. The levels of childhood TB (79.7%) in this study were high compared to other settings ranging from 2.1 to 19% [36–39]. One possible reason for this is that private providers may have recorded mostly already diagnosed child TB cases on the provided registers due to their workload constraints and they may have missed an unknown number of other presumptive TB cases. The levels varied among the districts, and this may reflect variations in completeness, with different compliances with reporting all “suspects”. Future research is recommended to further assess and verify these findings in the field.

## Conclusion

This study showed that many private health care providers rely on NTP supported laboratories for diagnosis, but they often do not report the children diagnosed with TB to NTP. The private health providers often rely on chest X-ray in addition to clinical symptoms for diagnosis TB in children. Communication between private providers, laboratory and NTP treatment centres could and should be strengthened through training.

## Data Availability

The datasets analyzed during the current study are not publicly available due to maintaining the confidentiality of participants keeping in view the ethical consideration for stigmatized infectious diseases i.e. TB, but are available from the corresponding author on reasonable request.

## Abbreviations

AJK: Azad Jammu & Kashmir
BCG: Bacillus Calmette–Guérin
GB: Gilgit Baltistan
KPK: Khyber Pakhtunkhwa
NTP: National TB Control Program
PPA: Pakistan Pediatric Association
TB: Tuberculosis
